# The Treatment of Aortic Valve Stenosis in Intermediate and Low-Risk Patients—When, How and Where

**DOI:** 10.3390/jcm11041073

**Published:** 2022-02-18

**Authors:** Flavio L. Ribichini, Valeria Ferrero

**Affiliations:** Division of Cardiology, Cardio-Thoracic Department, University of Verona, 37126 Verona, Italy; valeria.ferrero@aovr.veneto.it

## 1. When Treating a Severe Aortic Stenosis

Until now, treatment of severe aortic stenosis (AS) is recommended after the disease becomes symptomatic or causes “silent” damages such as myocardial dysfunction. Such behavior is the remaining heritage of a past when medicine had fewer alternatives and the timing of a surgical valve replacement was the main driver of a therapeutic approach that was viewed as a “*one chance in life*” option.

Aortic stenosis (or insufficiency) is not only a valve disease, or even only a heart disease, but should be seen as a systemic disease as with any other valvular heart disease. This is due to the fact that the reduction of cardiac output secondary to the valve dysfunction affects every single cell of the human body and the compensatory mechanisms that the heart implements to compensate affect the heart itself, causing hypertrophy, ischemia, fibrosis, loss of compliance, dilatation and a series of hemodynamic changes that, chained together, impact the hemodynamics of the whole human body.

Why then wait until aortic stenosis becomes severe if it is already known that symptoms may occur also with only “moderate” aortic stenosis? [1]. Surgeons in fact are already re-visiting their historical behavior and anticipating valve replacement [2]. Considering the detrimental effects of the compensatory changes imposed by a failing heart valve, a similar position could be applied to other valvular heart diseases, as it was suggested more than 10 years ago by Enriquez-Sarano in relation to the insufficiency of the mitral valve [3].

Today it is hard to conceive living with a severely stenotic aortic valve that can be replaced by a transcatheter heart valve (THV) in 30 min by a percutaneous intervention performed in local anesthesia. Some concerns may persist in terms of durability, or due to the fact that it is not clear what the next approach will be after THV. These are certainly open questions, but today, and until these questions are answered, should we still advise a patient with severe AS to wait?

Undoubtfully, a body with a well-functioning valve will perform better compared to one with a failing valve (although it is asymptomatic). Muscles contraction, liver metabolism, glomerular filtration, neuronal connections, alveolo-capillar exchange, cardiac chamber compliance, microvascular function—all will benefit from an optimized circulatory system. What to do when the THV will fail is a matter of research and development, and answers will be available soon. In the meantime, there is no robust evidence to advise an intermediate to low-risk patient to live with an untreated severely stenotic aortic valve.

## 2. How to Treat Aortic Stenosis 

In this supplement of the JCM dedicated to “Surgical or Transcatheter Aortic Valve Replacement in Intermediate-Risk Patients”, world-leading experts provide their vision on specific aspects of the management of AS stenosis in intermediate to low-risk patients. The different diagnostic and treatment modalities are explained and discussed. Surgery remains the first option in young and low-risk patients, in particular when AS is associated with other medical pathologic conditions such as the disease of the aortic root, other valvular defects, or severe forms of coronary artery disease. Transcatheter aortic valve implantation (TAVI) is the first option for the remaining population, particularly when the procedure can be performed percutaneously through the femoral route. Of note, a cornerstone of the management of patients with AS is the concept of the heart valve clinic and its referring network. Indeed, the patient’s care process starts when the General Practitioner (GP) identifies a suspected heart rumor, even before the onset of symptoms, and addresses the patient to specific examinations to properly stage the disease, assessing the patients’ general conditions. Once the patient is taken care of by a dedicated heart valve clinic, all future actions are systematically coordinated to offer the best therapy and avoid misleading steps, reducing costs, useless examinations, and, above all, reducing the risk of suffering the consequences of the disease. This precise pathway that links the patient from the GP’s office to the operating room is definitely one of the main and most revolutionary messages contained in the latest European Guidelines for the treatment of valvular heart diseases [4].

## 3. Where to Treat Patients with Severe Aortic Stenosis

Aortic stenosis is a very frequent disease, and its prevalence has been well defined in different ethnicities and geographic areas. It is estimated that 400 to 500 aortic valves should be repaired each year out of 1,000,000 inhabitants [5].

The outcomes of interventions, be it surgical or THV, are in direct relationship with the team and operator’s experience. Indeed, a recent analysis of the US availability of centers that perform TAVI interventions has shown worrisome results with an unacceptable increment of mortality after the opening of new THV centers that treat lower risk patients [6]. On the other hand, it appears obvious, and has been demonstrated several times, that outcome is a byproduct of experience, and although monitoring of outcome is historically common practice among cardiac surgeons, this seems to be a “new requisite” in the interventionalists world. Monitoring clinical outcome after interventions is not trivial; dedicated statistical methods should be applied, and the most widely accepted is the cumulative sum of failures method (CUSUM) [7]. There are few dedicated analyses using this method in the context of TAVI procedures, and one of the articles of this supplement is dedicated to this important aspect [8]. The study provides the largest analysis of this kind available in the literature, and clearly demonstrates that proficiency in performing TAVI in patients at intermediate to low risk is achieved after the performance of more than 450 TAVI procedures.

High-volume heart valves centers should therefore represent the preferred option for patient’s referral and spoke operators training and integration in a quality and safety-oriented environment.

As indicated by the guidelines, such procedures should be centralized in dedicated high-volume heart valve clinics where all of the necessary equipment and professionals are readily available in a system that warrants the best clinical outcomes at the most contained costs after the most efficient management of every single patient, irrespective of clinical risk. The involvement and active participation of GPs and cardiologist from spoke centers in the decision-making process, and training on diagnostic and therapeutic skills in a quality and safety-oriented environment, should represent a global organizational goal to expand in the future to all forms of advanced forms of cardiovascular interventions.
